# Re-irradiation of recurrent lung tumours: Associations between dose and 2-year survival^☆^

**DOI:** 10.1016/j.ctro.2025.101036

**Published:** 2025-08-21

**Authors:** Robert Rulach, Stephen Harrow, Anthony J. Chalmers, John Fenwick

**Affiliations:** a University of Oxford, Department of Oncology, Old Road Campus Research Building, Roosevelt Drive, Oxford OX3 7DQ, UK; b NHS Lothian, Edinburgh Cancer Centre, Western General Hospital, Crewe Rd S, Edinburgh EH4 2XU, UK; c School of Cancer Sciences, University of Glasgow, Glasgow G61 1QH, UK; d University College London, Department of Medical Physics & Biomedical Engineering, Gower St, London WC1E 6BT, UK

**Keywords:** Re-irradiation, Lung neoplasms, Intra-thoracic recurrence

## Abstract

**Background and purpose:**

Loco-regional recurrence is seen in up to 50 % of patients with non-small cell lung cancer (NSCLC) by 2 years after curative intent radiotherapy (RT). For these patients, radical re-irradiation (re-RT) is possible, but data is lacking regarding efficacy and radiation dose response, especially about conventionally fractionated re-irradiation. We analysed associations between survival following re-irradiation and RT dose, to guide clinicians regarding target re-RT dose, and predict re-treatment efficacy.

**Material and methods:**

We performed a literature search for studies primarily comprised of NSCLC patients that detailed 2-year overall survival (OS_2-yr_) rates and delivered doses. These data were collated with intervals between treatments, PTV sizes and use of concurrent chemotherapy where this information was available. Logistic regression analyses of associations between OS_2-yr_ and treatment and patient factors were carried out. Doses required for 30% and 50% OS_2-yr_ were calculated.

**Results:**

We identified 20 suitable studies (675 patients). In univariable models, OS_2-yr_ was significantly associated with the initial RT dose, re-RT dose and chemotherapy use but not the interval. The best multivariable OS_2-yr_ model according to the Akaike Information Criterion included only the re-RT dose (p < 0.05) and described the data well (Hosmer-Lemeshow p-value = 0.385). This model predicted OS_2-yr_ rates of 30 % and 50 % at re-RT equivalent doses in 2 Gy fractions (EQD2s) of 49.8 Gy_10_ (95 % CI 36.4, 58.0 Gy_10_) and 76.5 Gy_10_ (95 % CI 70.8, 82.7 Gy_10_) respectively.

**Conclusion:**

OS_2-yr_ following re-RT of recurrent NSCLC is significantly associated with retreatment dose. A reasonable target dose for re-RT is EQD2s > 50 Gy_10_ with survival rates continuing to increase to 85 Gy_10_.

## Highlights

• The relationship between the efficacy of re-irradiation (re-RT) for non-small cell lung cancer and the retreatment dose is poorly understood.
• We built a dataset with the 2-year overall survival rate (OSR) from re-RT and variables from published data to model the effect of re-RT dose on OSR.
• Initial dose, the re-RT dose and chemotherapy use were significantly associated with 2-year OSR on univariable logistic regression modelling.
• The model that best fitted the data included only re-RT dose and predicted 2-year OSR of 30 % and 50 % with re-RT doses of 49.8 Gy_10_ and 76.5 Gy_10_ EQD2.
• These results support giving a re-irradiation dose of > 50 Gy_10_ EQD2, or higher, whilst respecting cumulative organ at risk tolerances.

## Introduction

Local recurrence rates of up to 50 % have been reported for patients with non-small cell lung cancer (NSCLC) at two years after curative intent radiotherapy (RT), depending on disease stage and treatment factors [[1], [2], [3]]. Options for treating recurrences are limited. Surgery is difficult due to patient fitness and the higher risk of complications, and systemic treatment has significant side-effects.

Radical re-irradiation (re-RT) was first delivered to highly selected patients in the 1960 s. Recently, it has been used increasingly often in the treatment of thoracic tumours [[4], [5], [6]], although evidence for its efficacy consists mainly of retrospective reports with few prospective trials. Reported rates of 2-year overall survival (OS_2-yr_) following re-RT range from 11 % to 74 % [[7], [8]], reflecting the multiplicity of factors thought to influence survival. These include RT technique, dose, interval between radiation courses, amount of spatial overlap of the target volumes, use of chemotherapy, disease stage and tumour size. There are several expert re-RT consensus publications to aid patient selection and treatment given the limited data, but only one recommends a specific dose for re-RT [[9], [10], [11], [12]].

Toxicity rates are higher for re-RT than for primary RT [13], due to the cumulative normal tissue effects of the first and second RT treatments. Consequently, when considering re-RT, it would be useful to be able to predict the survival rate for a planned prescribed dose and how this would change if the dose was reduced to accommodate normal tissue constraints. There have been recent publications looking at stereotactic ablative body radiotherapy (SABR) re-irradiation alone but there is little data on conventional fractionation [[14], [15], [16]].

In this study we have therefore collated re-irradiation data for both SABR and conventionally fractionated re-irradiation and analysed relationships between OS_2-yr_ and factors including retreatment dose and the interval between treatments. Our aim is to build models that assist with patient selection, dose prescription and counselling about re-irradiation outcomes.

## Methods

### Data collection

We searched for re-RT studies published in English between 1st January 1970 and 1st December 2020 which reported outcomes for adult humans given two courses of RT for lung cancer using MEDLINE and the University of Glasgow search engine. The search strategy is detailed in the Appendix.

Studies included in the dataset treated ≥ 50 % of patients with NSCLC (rather than SCLC or metastases), reported doses for both RT courses and provided OS_2-yr_ rates. Cumulative dose to planning target volume (PTV) was either taken directly from the study or calculated using the prescription doses from the first and second treatment where it was reasonable to do so (i.e. with overlapping PTVs). The size of the target volume, the interval between treatments and the fraction of patients receiving concurrent chemotherapy (conCT) were also tabulated where available. Animal model data were excluded.

### Choice of endpoints and predictors

OS_2-yr_ was selected as the outcome of interest because it is clinically relevant and commonly reported. The predictor variables studied were the dose prescribed for the initial treatment, the retreatment dose, cumulative maximum dose (D_max_) to the PTV, interval between treatments, re-RT PTV size and use of conCT.

### Data processing

For each study, OS_2-yr_ and the median doses given at primary RT and at re-RT were converted to tumour equivalent doses in 2 Gy fractions (EQD2s) using the linear-quadratic model (α/β = 10). For non-photon re-RTs, the EQD2s were adjusted for relative biological effectiveness (RBE). If a study gave a measure of tumour size other than the PTV (e.g. gross tumour volume), it was assumed that the tumour was spherical and then was expanded based on the details in each study to estimate the PTV. This process is described in more detail in the Appendix.

### Corrections for limited follow-up

The OS_2-yr_ rates were calculated actuarially but were dependent on the length of follow-up. For studies that had completed 2 years follow-up, no adjustment was required. For studies with a shorter follow-up period, to account for censoring we replaced *N* for each study by a lower effective patient number *N_eff_*. This was derived from either the 95 % confidence interval if quoted, the Kaplan-Meier plot using the method described by Guyot *et al* [17], or estimated using a similar study (see Appendix for further technical details).

### Statistics and model fitting

Logistic regression analysis of the data was carried out by maximizing the likelihood

[1] $$L=\prod_{i}{{OS}_{2-yr-model,\;\;\;i}}^{\left(N_{\mathrm{eff},i}{\times \;OS}_{2-yr,i}\right)}\times {\left(1-{OS}_{2-yr-model,\;i}\right)}^{\left(N_{\mathrm{eff},i}\times \left(1-{\;OS}_{2-yr\;i}\right)\right)}$$

in which ${OS}_{2-yr,i}$, the observed survival in the cohort of patients belonging to the *i*th study, is modelled as

[2] $${OS}_{2-yr-model,\;\;\;i}=\frac{1}{1+exp\left(a-\sum_{f=1}^{F}b_{f}x_{f,i}\right)}$$

where $x_{f,i}$ is the cohort’s value for the *f*th of *F* modelled factors and *b_f_* is the regression coefficient for factor *f*.

Univariable OS_2-yr_ models were generated for each factor, assessing the significance of a factor’s association with survival by comparing the change in 2lnL with and without the factor to $\chi_{\left(1,p=.05\right)}^{2}$ = 3.84 (likelihood-ratio test). Factors with p-values < 0.2 in univariable models were then included in multivariable analyses. Multivariable model fits were obtained for comprehensive combinations of factors, and the model fit that best balanced was identified using the Akaike Information Criterion (AIC). The AIC is a measure that estimates how well a model will fit the data but also balances this goodness-of-fit against complexity. The lower the AIC, the better the model prediction.

Adequacy of data modelling was gauged using the Hosmer-Lemeshow test. This test groups the model predictions into deciles then compares the difference between the observed and expected frequencies of the outcome. A Hosmer-Lemeshow p-value > 0.05 indicates a better model fit. Model performance was visualized in calibration plots of the survival rates predicted for the various cohorts, graphed against observed rates.

Dose-levels corresponding to modelled 30 % and 50 % OS_2-yr_ rates were calculated from model fits. We determined 95 % CIs on these dose-levels by bootstrapping the data 2000 times, then refitting models to the bootstraps, finding the doses required for 30 % and 50 % OS_2-yr_ according to each bootstrap fit, and identifying 95 % CIs as the ranges of values obtained excluding the lowest and highest 2.5 percentiles. The bootstrapped datasets were created by initially labelling $N_{\mathrm{eff},i}\times {OS}_{2-yr,i}$ and $N_{\mathrm{eff},i}\times \left({1-OS}_{2-yr,\;i}\right)$ cases in each study *i* as being respectively alive and dead at 2 years, and then randomly resampling the case composition of each study with replacement.

The dataset was split into subgroups of studies categorized as treating patients with conventionally fractionated (‘Conv-Fr’), SABR (‘SABR-Fr’) or mixed radiation schedules. SABR schedules were defined as those giving doses-per-fraction > 5 Gy. OS_2-yr_ models were fitted to the subgroup data, to determine dose responses for the different schedule types considered separately. Using the AIC, the subgroup-based model’s ability to predict survival was compared to that of a single dose–response model fitted to the whole dataset. Finally, the process was repeated with the dataset split into groups of studies in which treated recurrences were all infield or a mix of infield and out-of-field.

Correlations between factors were characterized by Pearson *r* coefficients, with 2-sided significance assessed at the p = 0.05 level. All analyses were performed using R version 3.6.1 (R Foundation for Statistical Computing, Vienna, Austria).

## Results

### Data description and initial assessment

**Fig. 1:**
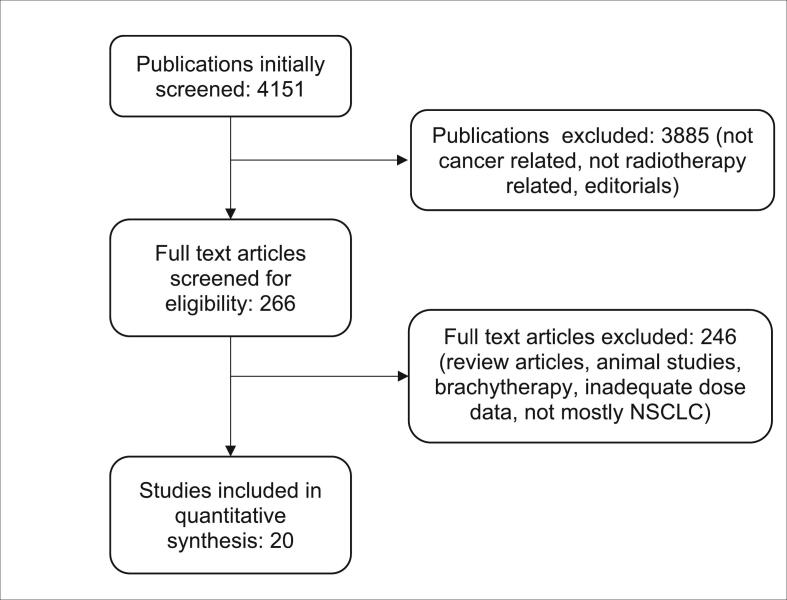
PRISMA diagram.

The literature search is described in Fig. 1. Data from the 20 studies identified are summarized in Table 1. Patient, treatment and follow-up details are listed study-by-study in Table A1 of the Appendix together with OS_2-yr_ rates, except for the studies of Griffioen *et al*^37^ and Tetar *et al*^38^ which we combined since they provided complementary data for overlapping patient groups, with the latter study including an additional six patients. Thus, we have analysed data for 19 patient cohorts.

**Table 1:** Dataset summary.

Data	Median value (range)^a^	Number of studies providing data	Total *N*^b^	Total *N_eff_*^b^
OS_2-yr_ (%)	42 (11, 74)	20	675	506
Initial dose^c^ (EQD2 Gy_10_)	65 (42, 133)	20	675	506
Retreatment dose^c^ (EQD2 Gy_10_)	83 (40, 126)	20	675	506
Cumulative D_max_^c^ (EQD2 Gy_10_)	141 (94, 216)	20	675	506
Inter-treatment interval (months)	17 (8, 36)	20	675	506
Concurrent chemotherapy rate	0.33 (0, 0.62)	7	263	191
Estimated PTV size (cc^d^)	133 (24, 237)	12	389	298

a. For OS_2-yr_ we list the median and range of the actuarial 2-year overall survival rates for each study. For the treatment and patient factors, we list medians and ranges of the individual studies’ median values, which can be found in Table A1.

b. Total *N* and *N_eff_* in studies that contributed data.

c. Initial and retreatment doses denote the doses prescribed to the initially treated and retreatment PTVs respectively. Cumulative D_max_ denotes the maximum cumulative dose in the re-irradiation PTV.

d. cc = cubic centimetres.

A total of 675 patients were treated in the 19 cohorts, corresponding to an effective number *N_eff_* of 508 patients after allowing for incomplete follow-up. The median OS_2-yr_ rate in the studies was 42 % and the range was 11–74 % with an average of 48 % when weighted by *N_eff_*. The tumour type treated at re-irradiation was NSCLC in 594 of the 675 patients (88 %). Nine studies reported results for re-irradiation solely of local recurrences; the other ten reported results for local recurrences, second primary lung cancers and metastases combined. Overall, local recurrences accounted for 487 of the 675 cases in the dataset (72 %). Median PTV and rates of conCT administration were reported for only twelve (n = 389, 58 %) and seven (n = 263, 39 %) studies respectively.

### Univariable regression analyses of OS_2-yr_ in the full dataset

Results of univariable analyses are shown in Table 2. OS_2-yr_ was significantly associated with the initial RT prescribed dose, retreatment prescribed dose, cumulative *D_max_* to the re-RT PTV, use of conCT and PTV size. The retreatment and cumulative D_max_ EQD2s were strongly correlated (Pearson *r* = 0.73, p = 0.00036) as shown in Supplementary Fig. 1. Correlations between the initial and retreatment EQD2s were weaker (*r* = 0.14, p = 0.58).

**Table 2:** Univariable analyses of associations between OS_2-yr_ and patient and treatment factors.

Predictor	N*_eff_^a^*	p-value	Sign of association
Initial dose*	506	0.029	+
Retreatment dose*	506	1.92x10^-10^	+
Cumulative D_max_*	506	2.42x10^-7^	+
Interval between treatments	506	0.824	n/a
Concurrent chemotherapy rate*	191	0.047	+
Estimated PTV size*	298	0.028	−

* Factors with p-values < 0.05.

a. Effective numbers of patients for whom data was available for each factor assessed.

**Fig. 2:**
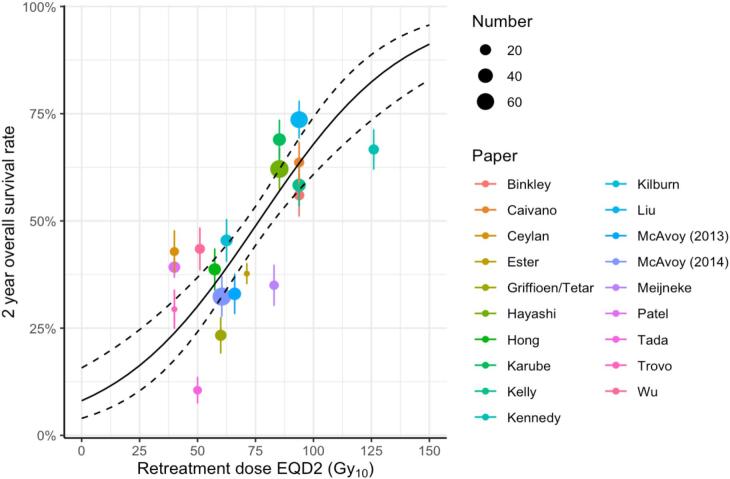
Univariable logistic regression of OS_2-yr_ against prescribed retreatment dose in the full 19 cohort dataset. The solid line is the fitted regression curve and the dashed lines show the standard error of the regression. The dots represent survival rates for each individual cohort with sizes proportional to effective patient numbers. Vertical bars are 68% confidence intervals, calculated from studies’ survival rates and effective patient numbers.

Fig. 2 shows the logistic regression of OS_2-yr_ against retreatment dose, which individually was the factor most significantly associated with survival. The regression has the form

**Fig. 3:**
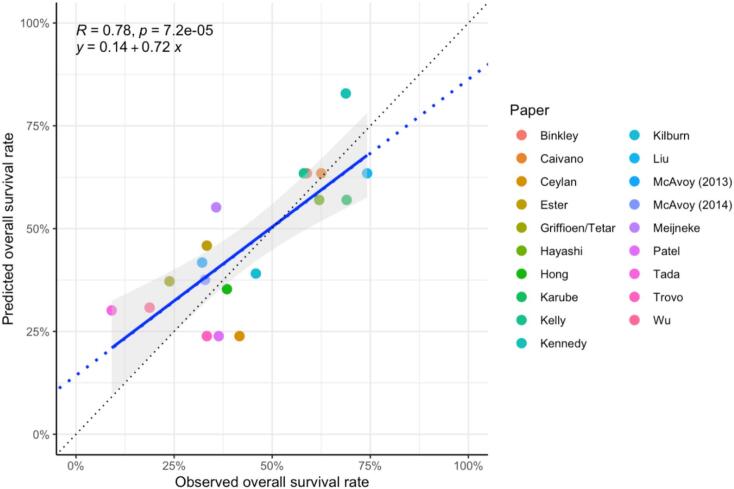
Calibration plot showing survival rates predicted by the univariable OS_2-yr_ model based on retreatment dose, graphed against the OS_2-yr_ rates observed in each cohort. The blue and black lines are the best linear fit (further detailed in the plot) and line of identity. (For interpretation of the references to colour in this figure legend, the reader is referred to the web version of this article.)

[3] $$p\left({OS}_{2yr}\right)=\frac{1}{1+e^{-(-2.5929\;\;+0.0318\;RD)}}$$

where *RD* is the retreatment dose in EQD2 Gy. The fit describes OS_2-yr_ rates of 30 % and 50 % at retreatment doses of 49.8 Gy_10_ (95 % CI 36.4, 58.0 Gy_10_) and 76.5 Gy_10_ (95 % CI 70.8, 82.7 Gy_10_) respectively, with a 0.6 % survival increase per 1 % increase in EQD2. The fIt’s Hosmer-Lemeshow p-value was 0.385, indicating a reasonable description of the data. There was a strong correlation between the OS_2-yr_ rates predicted and observed for each cohort [18] (Pearson *r* = 0.78, p < 10^-4^, Fig. 3).

### Multivariable regression analyses for OS_2-yr_

All the factors tested univariably were included in multivariable analyses, except for the interval between treatments which had a p-value ≥ 0.20 on univariable analysis. Only four studies provided data for both PTV size and conCT administration, and in these the two factors were moderately negatively correlated (Pearson *r* = -0.34, p = 0.66). We therefore built multivariable models from dosimetric data plus either one of these factors but not both. The two sets of models were tested separately in datasets comprising the twelve and seven cohorts respectively for where data were available. The dosimetric data included in the models comprised up to two of the initial, retreatment and cumulative doses, but not all three since any one is essentially defined by the other two. Thus, the goodness-of-fit and AIC values of models that included two of these dosimetric factors were independent of which two were selected.

The multivariable results are shown in Table 3. For the twelve cohorts for which PTV data were available, models that included the PTV all had higher (worse) AIC values than models based on dosimetric data alone, therefore PTV information did not improve models’ predictive ability. In this subset, models that included two dosimetric factors had marginally better AIC values than did the univariable model based on retreatment dose alone. However, in the full 19-cohort dataset the model based on retreatment dose alone had a marginally better AIC than did models that included two dosimetric factors (669.3 vs 670.5) and thus had the better predictive ability.

**Table 3:** Multivariable analysis of associations between OS_2-yr_ and patient and treatment factors. (a) Models that include PTV size, fitted to the 12 cohorts for which PTV data were available. (b) Models that include concurrent chemotherapy administration, fitted to the 7 cohorts for which chemotherapy data were available.

(3a) Models and predictors	Predictor p-values	AIC
*Three variable models*		
PTV size, initial dose, retreatment dose	0.51, 0.08, <0.01	392.9
PTV size, initial dose, cumulative D_max_	0.51, 0.10, <0.01	392.9
PTV size, retreatment dose, cumulative D_max_	0.51, 0.10, 0.08	392.9
*Two variable models*		
PTV size, initial dose	0.08, <0.01	402.8
PTV size, retreatment dose	0.62, <0.01	393.9
PTV size, cumulative dose	0.23, <0.01	393.8
Initial dose, retreatment dose,	0.09, 2.5x10^-4^	391.4
Initial dose, cumulative D_max_	0.05, 2.5x10^-4^	391.4
Retreatment dose, cumulative D_max_	0.05, 0.09	391.4
*One variable models*		
PTV size	0.08	412.4
Initial dose	0.0009	404.2
Retreatment dose	<0.0001	392.2
Cumulative dose	<0.0001	393.3

3(b) Models and predictors	Predictor p-values	AIC
*Three variable models*		
Chemotherapy, initial dose, retreatment dose	0.17, 0.61, 0.08	239.0
Chemotherapy, initial dose, cumulative dose	0.17, 0.35, 0.08	239.0
Chemotherapy, retreatment dose, cumulative dose	0.17, 0.35, 0.61	239.0
*Two variable models*		
Chemotherapy, initial dose	0.09, 0.01	240.2
Chemotherapy, retreatment dose	0.62, <0.01	237.2
Chemotherapy, cumulative dose	0.25, 0.02	237.8
Initial dose, retreatment dose	0.25, 0.01	238.8
Initial dose, cumulative D_max_	0.58, 0.01	238.8
Retreatment dose, cumulative D_max_	0.58, 0.25	238.8
*One variable models*		
Chemotherapy	0.05	241.5
Initial dose	0.15	243.5
Retreatment dose	<0.01	238.2
Cumulative dose	<0.01	237.2

**Fig. 4:**
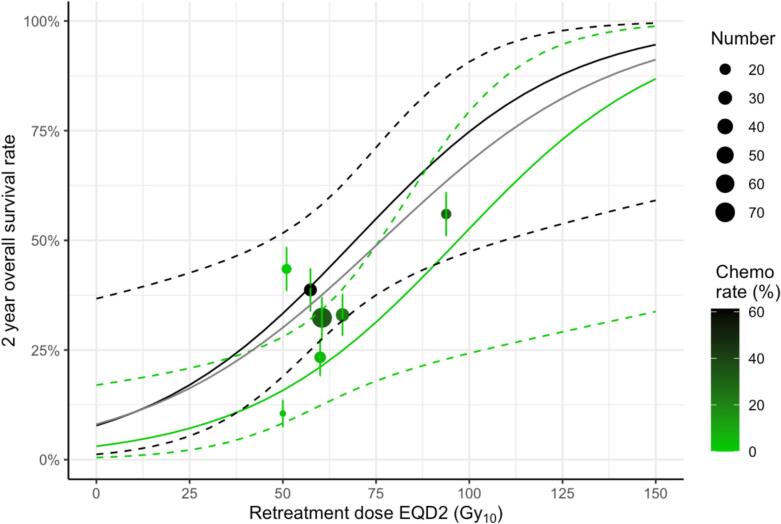
Plot of the multivariable OS_2-yr_ model based on retreatment dose and concurrent chemotherapy administration, fitted to the 7 cohorts for which chemotherapy data were available. The solid green and black lines show modelled dose–response curves for concurrent chemotherapy administration rates of 0% and 61% respectively. Dashed lines represent standard errors of the fit. Dots represent observed OS_2-yr_ rates in individual studies, with colours indicating concurrent chemotherapy administration rates. Vertical bars are 68% confidence intervals calculated from studies’ survival rates and effective patient numbers. For comparison, the grey line shows the dose–response of the univariable model of OS_2-yr_ versus retreatment dose fitted to the whole dataset. (For interpretation of the references to colour in this figure legend, the reader is referred to the web version of this article.)

In the 7-cohort conCT subset, a multivariable OS_2-yr_ model based on chemotherapy use and retreatment dose had the equal lowest AIC value of all models fitted. Fig. 4 shows OS_2-yr_ dose–response curves calculated for this model fit given chemotherapy administration rates of 0 % and 61 %, the lowest and highest rates in the studies. Observed OS_2-yr_ rates are also plotted in the figure, colour-coded by chemotherapy administration rates. There is a marked effect of conCT on the dose–response curves, with around 25 Gy_10_ less EQD2 being needed for a 50 % modelled OS_2-yr_ rate with a conCT rate of 61 % compared to 0 %. Despite this, there is no reason to prefer this model to a univariable model of OS_2-yr_ based on cumulative dose alone, which had an equally low AIC value. In turn, this univariable cumulative dose model performed less well in the full dataset than did the univariable model based on retreatment dose.

### Effect of schedule-type and location of recurrence

**Fig. 5:**
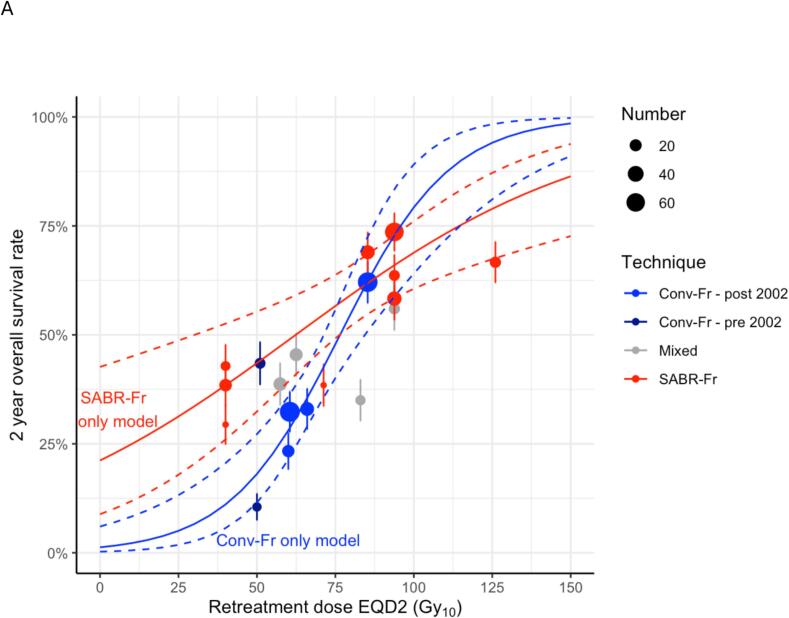

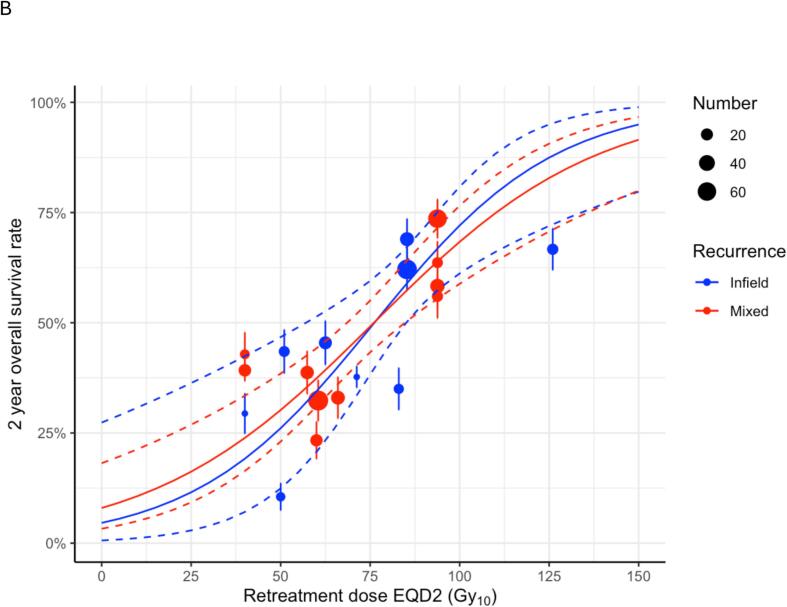
Plots of univariable models of OS_2-yr_ grouped by (A) schedule fractionation (Conv-Fr vs SABR-Fr vs mixed) and (B) recurrence type (infield vs mixed). Fitted dose-responses are shown as solid lines, while standard errors of the fits are represented as dashed lines. The dots describe the OS_2-yr_ rates observed for each cohort.

We explored whether the OS_2-yr_ dose–response was influenced by RT fractionation, beyond the dependence accounted for by calculating EQD2s via the linear-quadratic model with an *α*/*β* ratio of 10 Gy. The patient cohorts were split into three subgroups: Conv-Fr (6 cohorts, 302 patients, *N_eff_* = 220), SABR-Fr (9 cohorts, 264 patients, *N_eff_* = 207) and mixed (4 cohorts, 109 patients, *N_eff_* = 81). Separate univariable curves describing OS_2-yr_ versus retreatment dose were fitted to each subgroup. Compared to the univariable dose–response model fitted to the whole dataset, this subgroup-by-subgroup model included four additional fitted parameters. Its AIC was 671.0 compared to 669.3 for the single dose–response model fitted to the whole dataset, implying that it is a less parsimonious model. The individual dose–response curves fitted to Conv-Fr and SABR-Fr subgroup data are plotted in Fig. 5a. For all three models (Conv-Fr, SABR-Fr and the whole dataset), there is a significant dose–response relationship supporting dose escalation where possible, and the best model fit is to the whole dataset.

We also split the cohorts into subgroups that treated patients for infield recurrences only (9 cohorts, 270 patients, *N_eff_* = 200) or for mixed recurrences (10 cohorts, 404 patients, *N_eff_* = 308). Separate univariable curves of OS_2-yr_ versus retreatment dose were fitted for each subgroup and are shown in Fig. 5b. The AIC for the recurrence models was 671.3, worse than for the fit of the single dose–response curve to the whole dataset. Thus, the predictive ability of the modelling was not improved by fitting separate dose–response curves to the infield and mixed recurrence subgroups.

### Local control subset

**Local control data was available in a subset of 14 studies. On this limited dataset, PTV size and re-treatment dose were significant on multivariable modelling (see Appendix). Using a median PTV size of 112 cc, the dose required for 50 % rate of local control was 67.8 Gy_10_ EQD2 (95 % CI 49.43, 86.16).**

## Discussion

Re-RT of recurrent lung cancer raises complex clinical questions, with uncertainties regarding toxicity and efficacy, how best to balance these factors and to convey this to patients. There are several retrospective publications with small numbers (median number of patients in each study used in this analysis was 28 patients, range 13–102), therefore combining the data allows for much higher statistical power. An obvious concern is the degree that tumours that recur after RT might be radioresistant, perhaps making further RT futile. Contrary to this, our analysis indicates that recurrent lung cancers retain some radiosensitivity, and that higher re-RT doses, with either SABR or conventional fractionation, are significantly associated with increased OS_2-yr_ rate.

## Limitations

The patient cohorts studied in this retrospective analysis were heterogeneous and this potentially introduces confounding factors into the modelling. Although local recurrence was the main indication for re-irradiation in 72 % of patient cases, the other 28 % were a mixed group of local recurrences, second primary lung cancers (SPLCs), or metastases. Similarly, 88.6 % of patients had NSCLC, the others were re-irradiated for metastatic disease or lung cancers of different histology, and this might cause the dose–response found for the whole dataset to differ from that for re-irradiation of NSCLC alone. There was no significant difference between the dose–response curves fitted to the infield (i.e. local recurrences) and outfield/mixed recurrence (more likely to be SPLC) cohorts. Nevertheless, the lack of data regarding PTV overlap is a potential confounder, as it is likely that the re-treatment dose in those patients with no overlap is higher, and because they are SPLC, their response (and subsequent survival) may be better. Access to data at the individual patient level would allow these possible variations in dose–response to be more fully analysed.

PTV size and rate of conCT administration were not significantly associated with OS_2-yr_. However, data were missing for 42.4 % and 61.0 % of patients for PTV size and chemotherapy rate respectively. Therefore, significant associations might have been found if more data had been available. To get as much information as possible from the studies, we used standard expansions to derive the PTV from the GTV. This is a potential source of error, as every centre has different expansions and these will be different depending on the fractionation used. Unfortunately, the GTV was not consistently reported therefore any conclusions regarding the size of the tumour should be interpreted with caution.

Interestingly the interval between treatments was not significantly associated with OS_2-yr_ despite data being available for all cohorts in the dataset. Too little data was reported for patient fitness, comorbidities and the overlap between initially treated and recurrent tumour volumes to permit meaningful analysis of associations between these factors and OS_2-yr_.

The dataset included results for patients treated with photons, protons and carbon ions using conventional and SABR fractionated schedules. Of the seven studies that reported 2-year OS rates > 50 %, five gave SABR-Fr photon retreatments with median doses of 50 Gy in four or five fractions [8,[19], [20], [21], [22]] and two gave Conv-Fr [[23], [24]] or SABR-Fr [[23], [24]] carbon ion re-treatments. Our subgroup analysis of Conv-Fr and SABR-Fr re-irradiation found that retreatment dose was significantly associated with survival for each subgroup (Fig. 5a), although the overall model better predicted the data. Nevertheless, the apparent trend for improved survival from SABR-Fr may be due to confounding by indication, because SABR-Fr is generally used for small recurrences with no nodal spread whereas Conv-Fr is used for larger recurrences with nodal involvement, and nodal positivity was not included in the analysis as a predictive factor since data was not reported sufficiently often. Alternatively, high doses-per-fraction given in SABR treatments may be more effective than conventionally fractionated radiation, even after allowing for linear-quadratic effects with an α/β value of 10 Gy.

Before 2002, patients with early-stage disease may have been treated with Conv-Fr, whereas after this year early-stage cancers were treated increasingly often using SABR. Thus, it is possible that better survival rates would have been reported for Conv-Fr studies carried out before rather than after 2002. However, no difference was seen in our dataset.

An underlying limitation of our study is the endpoint analysed, OS_2-yr_. While this endpoint is clinically relevant and commonly reported, the local control rate would provide a more direct definition of re-irradiation efficacy, since it is not influenced by further treatments given following re-irradiation. The studies that this analysis is based on gave no further information regarding the subsequent lines of treatment patients after re-irradiation. This could have a significant influence on the results, especially given that there is a high risk of distant metastases – a recent phase II study of re-irradiation quoted a 2-year distant relapse rate of 47 % [25]. However, it should be noted that the studies reported patients largely treated between 2005 and 2015 where there were fewer systemic treatment options, and largely before immunotherapy, so the influence of systemic treatment if given on OS_2-yr_ may only be modest.

We analysed a subset of the dataset to look at local control and found that the retreatment dose and PTV size were significant on multivariable modelling. However, these results may be prone to confounding as PTV size is likely to be smaller in patients treated with SABR. Additionally, local control as an endpoint is difficult to assess as there is significant fibrosis post-RT and results depend on the frequency of surveillance scans. Nevertheless, both the OS_2-yr_ and the local control model show that the outcomes from re-irradiation are better with higher re-treatment dose. This finding may be confounded by several other factors such as size and location of tumour but is biologically rational and this finding is consistent with other published data.

### Comparison to other studies

Some retrospective studies have reported that outcomes are better at higher re-irradiation doses. Four studies show a trend for significantly improved overall survival and better local control when the re-RT dose is > 57–60 Gy_10_ EQD2 [[26], [27], [28], [29]]. In a recent prospective study, Dujim *et al.* have reported OS_2-yr_ rates of 52 % and 62 % respectively for patients (51 NSCLC, 8 SCLC, 1 unknown) treated using Conv-Fr with a median EQD2 of 60 Gy_10_ versus SABR-Fr with a median EQD2 of 77 Gy_10_ in a prospective study of re-irradiation for lung cancer [30].

There have been two *meta*-analyses of SABR re-irradiation data. The analysis presented in this paper is the first to also model conventionally fractionated re-irradiation. Our SABR data is consistent with the previous publications. Wang *et al.* performed logistic regression modelling of results from 12 SABR re-irradiation studies comprising a total of 195 patients and predicted a 50 % rate of OS_2-yr_ rate for a re-irradiation dose of 41.6 Gy in 5 fractions (63.5 Gy_10_ EQD2, γ_50_ 0.81) [16]. Viani *et al.* performed a *meta*-regression analysis of 19 studies (569 patients) and found that survival was significantly associated with tumour size, interval between treatments and re-irradiation dose [15]. The current analysis, modelling data from both conventional and SABR re-irradiation, demonstrates that increased dose is associated with better survival with any fractionation type, accepting that compromises are likely due to organs at risk tolerances, and concurrent chemotherapy may improve OS_2-yr_ rates.

### Clinical relevance and future work

Previous consensus guidelines recommended delivery of radical doses where possible [10] (BED > 100 Gy if using SABR, or 60 Gy in 30 fractions using CFRT). Our analysis supports this, with a modelled OS_2-yr_ rate of 37.2 % for a re-irradiation dose of 60 Gy EQD2. Moreover, the modelling results suggest that, when safely deliverable, higher tumour doses may offer improved OS_2-yr_ in this patient group, with an increase of 7.7 % absolute in modelled OS_2-yr_ per 10 Gy increase in EQD2 beyond 60 Gy_10_. Due to its high degree of conformality and associated dose sparing of organs at risk, SABR may be the preferred re-irradiation treatment in low-volume recurrences as it allows for a higher dose. For patients unsuitable for SABR, due to larger volume or central disease, there is a need for better treatments, with the possibility of adding radiosensitizers or immunotherapy, as explored in a recent trial of re-irradiation followed by adjuvant pembrolizumab [31].

There has been significant developments over the past decades in improving the sparing of normal tissues, and preventing consequential late toxicity. This is both important in the setting of primary radiotherapy and at re-irradiation. The advances in image guidance, breath-hold or gated treatments, volumetric arc therapy, and adaptive therapy using MR linac both facilitate re-irradiation now but also mean that patients having primary radiotherapy with these techniques, may have better re-irradiation options in the future [32].

Alongside demonstrating a dose–response, safe and efficacious dose escalation of retreatments requires the development of dose-volume models of toxicity, to guide trade-offs between normal tissue and tumour irradiation. Our group have further analysed the published literature to address this issue. Prospective studies are required to provide patient-level information to confirm and refine the results we have obtained, and to develop a translational research pipeline in recurrent disease.

## Declaration of competing interest

The authors declare that they have no known competing financial interests or personal relationships that could have appeared to influence the work reported in this paper.
