# Supplementary material for: Re-irradiation of recurrent lung tumours: Associations between dose and 2-year survival
Source: Clin Transl Radiat Oncol. 2025 Aug 21;55:101036. doi: 10.1016/j.ctro.2025.101036 (PMC12398877; doi:10.1016/j.ctro.2025.101036)

**Appendix**

**Sections:**

1. **Additional data regarding methodology**
2. **Additional data regarding modelling results**
3. **Putative local control model**
4. ***Methods***

Additional information regarding the precise methodology of data collection, processing and correction for limited follow-up is detailed below.

*Details of search strategy*

A literature search was conducted using MEDLINE and the University of Glasgow search engine, identifying any English language studies from 1st January 1970 to 1st December 2020 which included adult humans who had two courses of radiotherapy for lung cancer, where both the dose given to the tumour and the outcome data were published. Animal models were excluded.

The MEDLINE search strategy was ((lung AND cancer) OR non-small cell lung cancer) AND (retreatment OR re-treatment OR re-irradiation OR reirradiation).

*Data processing*

For each study, OS_2-yr_ and the median doses given at primary RT and at re-RT were converted to tumour equivalent doses in 2 Gy fractions (EQD2s) using the linear-quadratic model. For non-photon re-RTs, the EQD2s were adjusted for relative biological effectiveness (RBE). If a study gave a measure of tumour size other than the PTV (e.g. gross tumour volume), it was assumed that the tumour was spherical, and then was expanded based on the details in each study to estimate the PTV. This process is described in more detail in the Appendix.

For each study, OS_2-yr_ and the number of patients treated, N, were noted. Median doses prescribed at primary RT and re-irradiation were converted to tumour equivalent doses in 2 Gy fractions (EQD2s) using the linear-quadratic model:

$EQD2= D\frac{\left( d+\frac{\alpha}{\beta} \right)}{\left( 2+\frac{\alpha}{\beta} \right)}$ [1]

where D is the total dose given in an RT course, d is the dose-per-fraction and a 10 Gy tumour α/β value was used. For proton and carbon ion treatments, EQD2s were multiplied by relative biological effectiveness (RBE) values detailed in study publications, to correct them for the change in modality.

For some studies, the Intervals between the two RT treatments, use of concurrent chemotherapy at re-RT and target volume sizes were not reported and were marked up as missing. When PTV size was reported, the median value for a study was added to the database without modification. When gross, clinical or internal target volumes (GTV, CTV, ITV) were reported instead, PTV sizes were estimated by first calculating a target’s radius from its volume assuming sphericity, then increasing the radius by the GTV-PTV, CTV-PTV or ITV-PTV margin specified for the study, and finally calculating the volume of the PTV from the increased radius.

*Corrections for limited follow-up*

OS_2-yr_ rates reported by the re-irradiation studies had been calculated actuarially, censoring at last follow-up those patients who were not followed-up until the full 2 years or death. This diminishes the statistical precision of the calculated rates compared to levels expected based on binomial statistics, according to which the 1 standard error uncertainty on OS_2-yr_ in a cohort of *N* patients is:

$\sigma_{\mathrm{OS}_{2-yr}}= \sqrt{{\mathrm{OS}_{2-yr}\left( 1-\mathrm{OS}_{2-yr} \right)}/N}$ [1]

To fit the survival data, we used a maximum-likelihood method that assumes the precision of survival rates follows equation (1), as described in the *Statistics and model fitting* section. To account for the reduced precision caused by patient censoring we replaced *N* for each study by a lower effective patient number *N_eff_*. For studies that provided a 95% confidence interval (CI) on OS_2-yr_, we calculated *N_eff_* as:

$N_{eff}=\frac{\mathrm{OS}_{2-yr}(1 -\mathrm{OS}_{2-yr})}{{(95\% \mathrm{CI}/3.92)}^{2}}$ [2]

where the 95% CI was understood to cover a range of ±1.96 standard errors.

When a 95% CI was not reported but a Kaplan-Meier survival plot was available, the plot was digitized using online software (https://apps.automeris.io/wpd/) and reconstituted into a life-table using the R algorithm described by Guyot *et al*^14^. Then the 95% CI for OS_2-yr_ was calculated from the life-table, allowing *N_eff_* to be obtained via equation [2]. When neither a 95% CI nor a Kaplan-Meier plot were reported for a study, *N_eff_* was estimated by multiplying the total number of patients by the (*N_eff_*/*N*) ratio found for another study with similar follow-up.

1. **Results**

*Table A1. Details of the dataset analysed, which comprised 20 studies grouped into 19 cohorts, reporting results for 675 patients.*

| Study | Re-treat^a^ fractionation | Tumour type (NSCLC, SCLC, Other, %) | Infield recurrence  (%) | *N* | *N_eff_* | Median re-treat dose (EQD2 Gy_10_,  *α/β* =10) | Re-treat modality | Median  interval (months,  range) | OS_2-yr_ (%) | Follow-up  post re-treat (months, range) | Subsequent lines of treatment |
| --- | --- | --- | --- | --- | --- | --- | --- | --- | --- | --- | --- |
| Tada^7^ | Conv-Fr^b^ | 100, 0, 0 | 100 | 19 | 12 | 50 | Photon | 16 (5-60) | 11 | NR^c^ | NR |
| McAvoy^30^ | Conv-Fr | 100, 0, 0 | 63.6 | 33 | 28 | 66 | Proton (passive scatter) | 36 (2-376) | 33 | 11 (1-32) | NR |
| McAvoy^31^ | Conv-Fr | 100, 0, 0 | 53.9 | 102 | 73 | 61 | Photon or proton | 17 (1-376) | 33 | 7 (0-72) | NR |
| Griffioen^32^/ Tetar^33^ | Conv-Fr | 100, 0, 0 | 53.3 | 30 | 21 | 60 | Photon | 30 (5-189) | 23 | 25 | NR |
| Hayashi^20^ | Conv-Fr | 76.8, 0,  23.2 mets | 100 | 95 | 70 | 85 | Carbon | 17 (6-139) | 62 | 18 (1-89) | Reported for 1 pt |
| Wu^34^ | Conv-Fr | 69.5, 30.5, 0 | 100 | 23 | 16 | 51 | Photon | 13 (6-42) | 42 | 15 (2-37) | NR |
| Kennedy^16^ | SABR-Fr | 100, 0, 0 | 100 | 21 | 17 | 126 | Photon | 23 (7-52) | 68 | 24 (3-60) | NR |
| Trovo^35^ | SABR-Fr | 100, 0, 0 | 100 | 17 | 9 | 40 | Photon | 18 (1-60) | 29 | 18 (4 - 57) | NR |
| Karube^21^ | SABR-Fr | 100, 0, 0 | 100 | 29 | 29 | 85 | Carbon | 20 (8-99) | 69 | 29 (4-88) | NR |
| Ceylan^36^ | SABR-Fr | 100, 0, 0 | 64.3 | 28 | 13 | 40 | Photon | 14 (4-56) | 42 | 9 (3-93) | NR |
| Kelly^19^ | SABR-Fr | 94.4, 0,  5.6 other | 33.3 | 36 | 31 | 94 | Photon | 22 (0-92) | 59 | 15 (4-45) | NR |
| Patel^37^ | SABR-Fr | 88.5, 0,  11.5 other | 92.3 | 26 | 22 | 40 | Photon | 8 (3-26) | 37 | NR | NR |
| Ester^38^ | SABR-Fr | 83.3, 8.3,  8.3 Wilms | 100 | 13 | 9 | 71 | Photon | 29 (5-85) | 36 | 11 (2 - 38) | NR |
| Liu^8^ | SABR-Fr | 79.2, 6.9, 13.9 other | 26.4 | 72 | 61 | 94 | Photon | 21 (0-106) | 74 | 16 (4-56) | NR |
| Caivano^17^ | SABR-Fr | 54.5, 4.5, 40.9 mets | 77.8 | 22 | 16 | 94 | Photon | 18 (6-66) | 63 | 13 (13 -65) | NR |
| Binkley^18^ | Mixed | 100, 0, 0 | 47.3 | 25 | 17 | 94 | Photon | 16 (1-71) | 57 | 17 (3-57) | NR |
| Hong^23^ | Mixed | 77.4, 22.6, 0 | 74.2 | 31 | 26 | 57 | Photon | 15 (4-56) | 39 | 17 (5-77) | NR |
| Kilburn^39^ | Mixed | 75.8, 12.1, 12.1 mets | 100 | 33 | 24 | 63 | Photon | 18 (6-61) | 45 | 17 (range NR) | 4 pts had further radiation |
| Meijneke^40^ | Mixed | 75, 10,  15 mets | 100 | 20 | 14 | 83 | Photon | 17 (2-33) | 33 | 12 (2-52) | NR |

a. Re-treat = re-irradiation.

b. Fr = fractions.

c. All patients had died at the time of reporting. NR = not reported.

d. Mets = metastases.

*Figure A1. Plots of (a) cumulative EQD2 versus retreatment EQD2, (b) initial EQD2 versus retreatment EQD2. Pearson r coefficients and associated p-values are shown.*

(a)


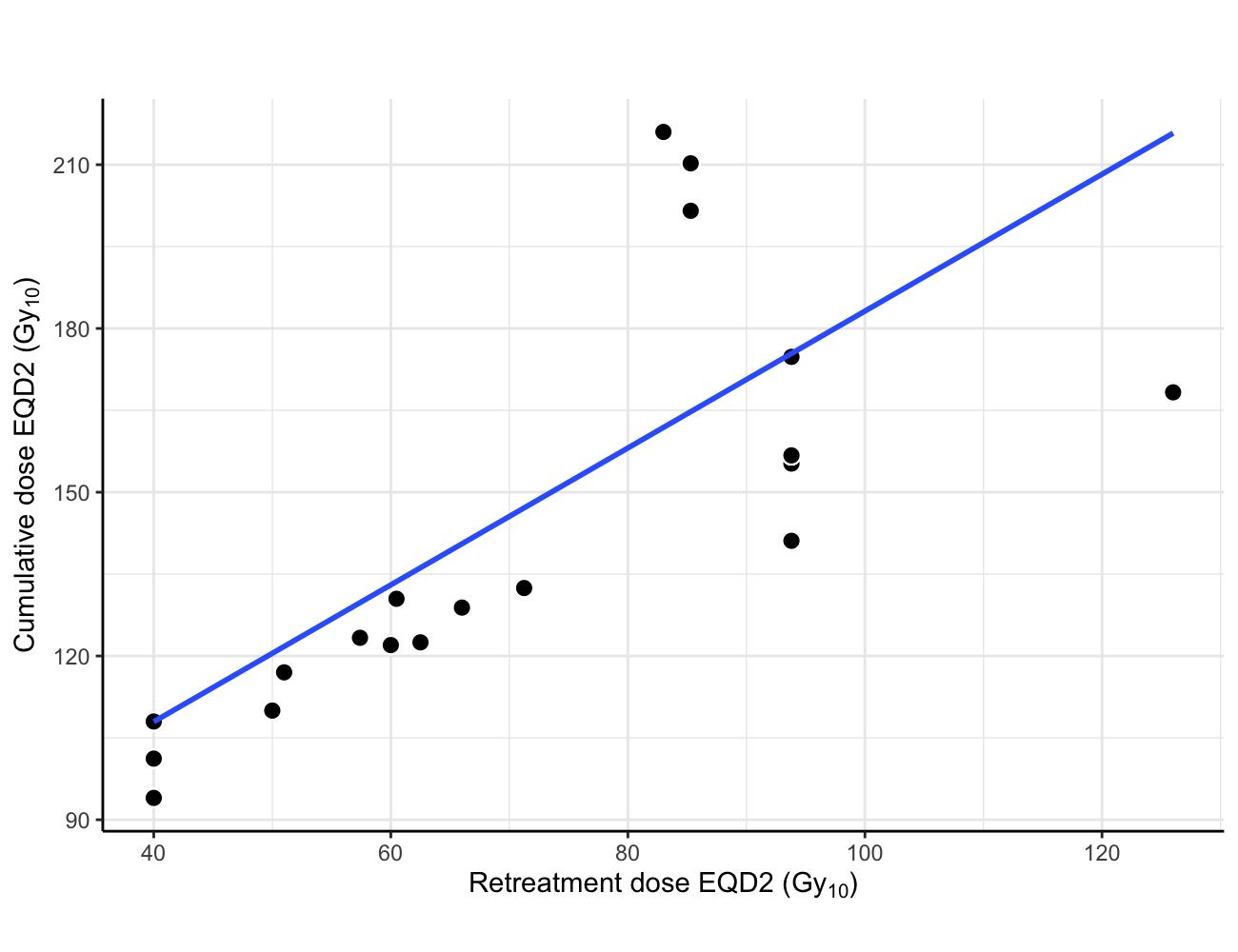


R= 0.73, p=0.00036

(b)


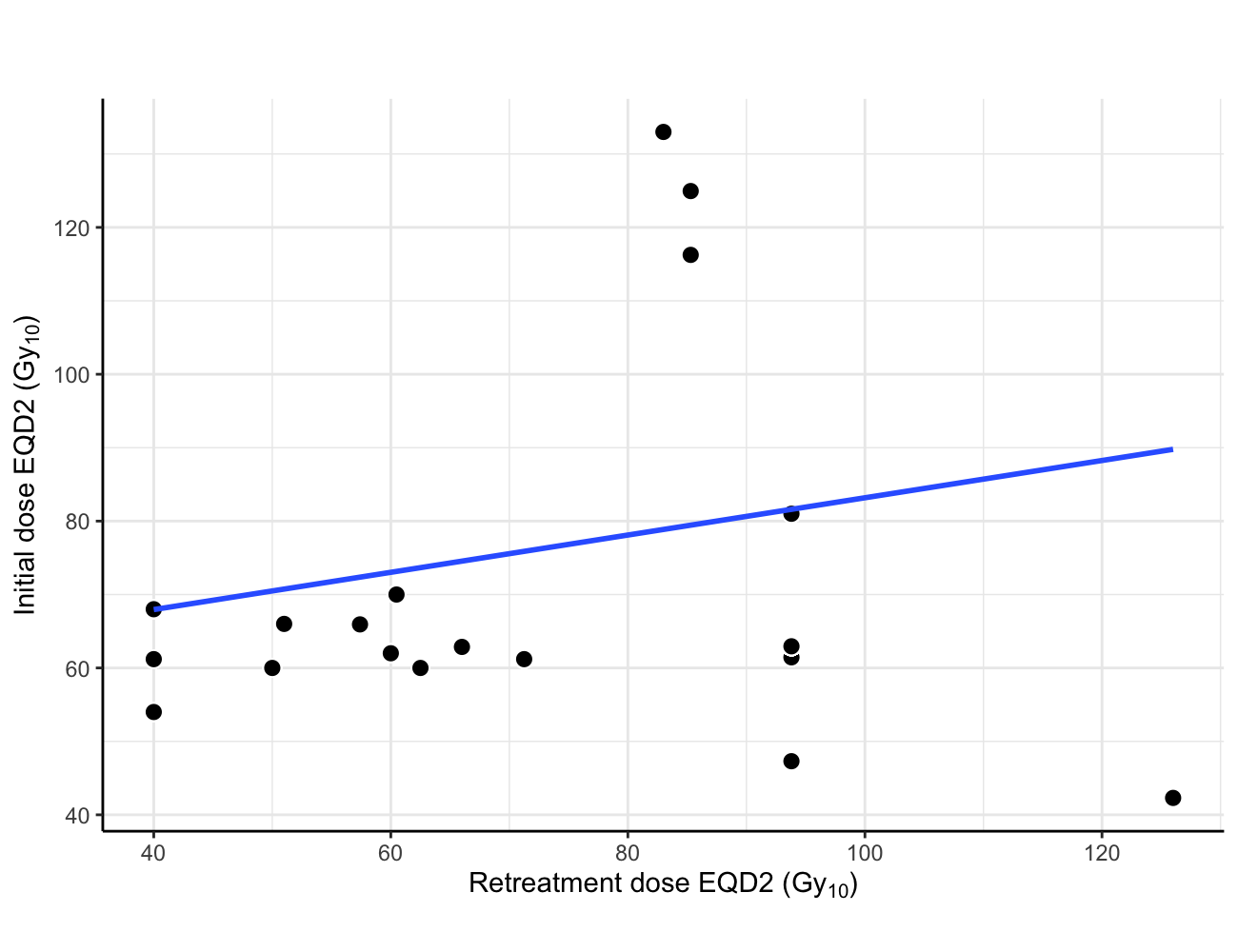


R= 0.14, p=0.58

1. **Putative Local Control Model**

Using a subset of the above 20 studies dataset, we identified 14 studies (n=502) that quoted the 2-year local control rates. These data are summarised in Table A2.

*Table A2 Summary of the local control subset*

|  |  | Missing values |
| --- | --- | --- |
| Number of trials: | 14 |  |
| Number of patients: | 502 |  |
| Effective number of patients at 2 years: | 297 |  |
| LC at 2 years (%) | 166 (55.9) | 0 |
| Local recurrence (%) | 131 (44.1) | 0 |
| Median interval from initial treatment and re-irradiation (months, range) | 17 (8 – 36) | 0 |
| Concurrent chemotherapy rate | 0.33 (0 – 0.613) | 186 |
| Median cumDmax to PTV (EQD2 Gy, range) | 155.2 (101.2 – 216) | 0 |
| Median initial treatment dose (EQD2 Gy, range) | 70 (42.3 – 133) | 0 |
| Median retreatment dose (EQD2 Gy, range) | 85.3 (40 – 126) | 0 |
| Median estimated PTV size (cc, range) | 112 (19.2 – 234.5) | 67 |

On univariable modelling, RT dose and PTV size were significant, and remained so on multivariable modelling (Table A3).

*Table A3 Summary of the local control univariable and multivariable models*

| Predictor | Local control | n | P-value |
| --- | --- | --- | --- |
|  | Univariable modelling results | | |
| Initial dose | 2-year LC | 297 | 0.421 |
| RT dose | 2-year LC | 297 | <0.001* |
| Interval | 2-year LC | 297 | 0.852 |
| PTV size | 2-year LC | 230 | <0.001* |
| Concurrent chemo rate | 2-year LC | 111 | 0.077 |
|  | Multivariable modelling results | | |
| PTV size | 2-year LC | 230 | 0.030* |
| RT dose | 2-year LC | 230 | 0.052 |

The multivariable model expression is:

P(2 year local control|X_1_,X_2_) = Φ(-0.7542 - 0.0048X_1_ + 0.0190X_2_)

Where X_1_ = estimated PTV size and X_2_ = retreatment dose.

The model uses information from 9 studies, a total of 230 patients. Using the median retreatment dose at 85.3Gy EQD2, the model predicted a 50% rate of 2-year local control for a PTV of 181.55cc (95%CI 88.40 – 274.70), and a 30% rate of 2-year local control for a PTV of 358.46cc (95% CI 120.2 – 596.72). The RT dose predicted to give a 50% 2-year local control rate (assuming the median PTV of 112cc) was 67.8Gy (95% CI 49.43, 86.16). This was bootstrapped 2000 times and the 95% CI was (-404.91, 569.94Gy). For 30% local rate, the predicted RT dose was 23.29 (95% CI -34.91, 81.49) and the bootstrapped 95% CI was (-508.97, 857.24). The model is plotted in Figure A2 with the source data and standard error of the regression.

*Figure A2. Plot of the local control multivariable model (estimated PTV and re-treatment dose).*

*The blue line is the fitted regression model, black dotted lines represent the standard error of the regression, the red dotted line indicates the 50% local control rate. The dots represent the toxicity rate from each individual paper coded by colour, with the size of the dots proportional to the number of patients in the study, vertical bars are the 68% binomial confidence interval. There are only 9 data points included in the plot five of the 14 studies lacked PTV data and were excluded from the multivariable model. This model uses the median PTV of 112cc.*


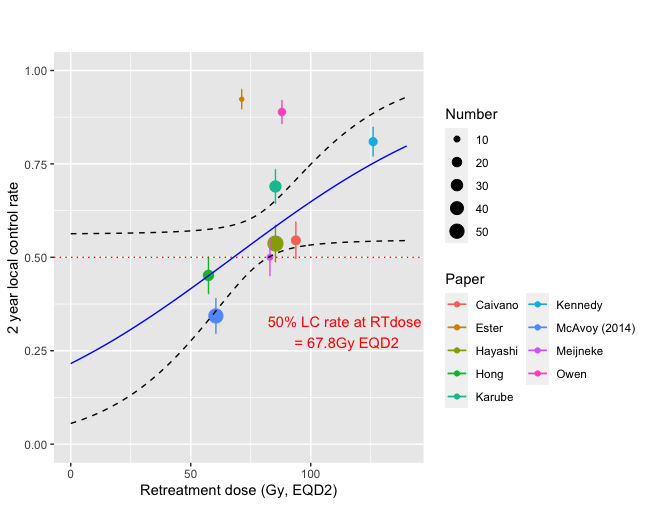

Supplement: Supplementary Data 1 [file mmc1.docx]
